# Eligibility Assessment for Intravenous Thrombolytic Therapy in Acute Ischemic Stroke Patients; Evaluating Barriers for Implementation

**DOI:** 10.5812/ircmj.11284

**Published:** 2014-05-05

**Authors:** Hormoz Ayromlou, Hassan Soleimanpour, Mehdi Farhoudi, Aliakbar Taheraghdam, Elyar Sadeghi Hokmabadi, Rouzbeh Rajaei Ghafouri, Mehdi Najafi Nashali, Ehsan Sharifipour, Somayeh Mostafaei, Davar Altafi

**Affiliations:** 1 Department of Neurology, Tabriz University of Medical Sciences, Tabriz, IR Iran; 2 Department of Emergency, Tabriz University of Medical Sciences, Tabriz, IR Iran

**Keywords:** Stroke, Thrombolytic Therapy, Developing Country, Eligibility Determination

## Abstract

**Background:**

Intravenous thrombolysis is an approved treatment method for patients with acute ischemic stroke (AIS) and is recommended by multiple guidelines. However, it seems that it is less frequently used in the developing countries compared to the developed countries.

**Objectives:**

The purpose of this study was to estimate the percentage of patients with AIS, eligible for intravenous thrombolytic therapy, at the main referral center in Northwest Iran and to determine the main barriers for implementation of this method.

**Patients and Methods:**

Over one year, 647 patients who were admitted to the emergency department and met the Cincinnati Stroke Scale were enrolled into the study. The center to which patients were admitted, is a tertiary university hospital that has the required infrastructure for thrombolytic therapy in AIS. Factors recorded were neurological examinations and time between onset of symptoms and hospital arrival, hospital arrival and performance of brain computed tomography (CT) scanning, and hospital arrival to complete the investigations. Patients eligible for intravenous thrombolytic therapy were identified according to the American Heart Association (AHA) guidelines.

**Results:**

Mean time interval between hospital arrival and completion of brain CT scanning was 91 minutes (range: 20–378 minutes) and mean time from hospital arrival to completion of investigations was 150 minutes (range: 30–540 minutes). A total of 159 (31.3%) patients arrived at hospital within 3 hours of the onset of symptoms (early enough for intravenous thrombolytic therapy). However, 81.7% (130/159) of these patients missed thrombolytic therapy due to delayed performance of brain CT scanning and laboratory tests and 38.3% (61/159) had contraindications. The remaining 16 patients (10% of those who arrived within 3 hours and 3.1% of all cases) were eligible for thrombolytic therapy.

**Conclusions:**

The major barriers for thrombolytic therapy for patients with AIS in this setting were delays in the provision of in-hospital services, like initial patient assessment, CT scans or laboratory studies. These results were in contrast with previous reports.

## 1. Background

Cerebrovascular disease is the second most common cause of death worldwide. It ranks in the sixth place for disease burden and it is expected to rise to fourth place by 2020 (1). For middle-income countries, cerebrovascular disease is the first leading cause of death and the third leading cause for burden of disease (2). Intravenous thrombolysis is an approved therapy for patients with acute ischemic stroke (AIS) (3) with approved safety and efficacy (4-6). Over 80% of all stroke deaths, worldwide, occur in the developing countries (7). Adults in low- and middle-income countries are at a 30% greater risk of death from non-communicable diseases than their counterparts in high-income countries (8), nonetheless, in developing countries thrombolytic therapy for patients with AIS is available for only 1–3% of patients and mostly in urban areas (9-13), while in developed countries, this ratio is as high as 10% (14) or even higher (15). Iran is a developing country with stroke prevalence of 23–103 per 100,000 population (7, 13). The country has 14 hospitals with the needed infrastructure for thrombolytic therapy (10), however, few centers are actually providing this treatment for patients.

## 2. Objectives

Our aim was to estimate the percentage of patients with AIS, eligible for intravenous thrombolytic therapy at the main tertiary referral center in Northwest Iran and to determine the main barriers for this therapy implementation.

## 3. Patients and Methods

This cross-sectional study was conducted in Imam Reza Medical Center, a tertiary referral university hospital. The center covers an urban population of more than 1.5 million in addition to one million rural population, with an annual admission of 120,000 patients. This complex has the required infrastructures for implementing thrombolytic therapy. Over a one-year study period, 647 patients, who met the Cincinnati stroke scale, with sudden onset of at least one of the following symptoms were enrolled into the study: facial droop, motor arm weakness or speech abnormalities. Information was recorded about the patients’ demographic profile, stroke risk factors, vital signs, time between symptoms onset and hospital arrival, hospital arrival and performing brain computed tomography (CT) scanning and hospital arrival and complete investigations and baseline CT findings. The time between symptoms onset and hospital arrival was defined as the last time patient had been seen healthy or the time of stroke symptom onset. The time between hospital arrival and performing brain CT scanning was defined as the interval between the times recorded on emergency department reception note and a hard copy of the CT scan. The time between hospital arrival and complete investigations was defined as the time interval between the emergency department reception note and a neurology resident fulfilling the contraindication form for thrombolytic therapy. CT scanning was available 24 hours a day, using a helical CT scanner.

Patients were classified as eligible if they had no contraindication for thrombolytic therapy and it was possible to initiate their treatment within three hours of the symptom onset. Patients with any contraindications for thrombolytic therapy, according to American Heart Association/American Stroke Association (AHA/ASA) guidelines (3), or a time of more than three hours from symptoms to needle, were classified as ineligible.

The proportion of patients, eligible was determined. The study did not consider the ability of patients or their family to pay for tissue plasminogen activator (TPA). Iran’s health insurance system does not cover the cost of TPA and the patients were not asked to give informed consent because they were not going to receive thrombolytic therapy. This study was approved by the Ethical Committee of Tabriz University of Medical Sciences. The data were analyzed using SPSS version 18.

## 4. Results

Over a one-year period, we enrolled 647 patients with at least one of the three abnormalities, determined using the Cincinnati Stroke Scale. Out of 647 patients, 115 patients (17.9%) had an intracerebral hemorrhage (ICH). After laboratory investigations and CT scanning, 515 (79.4%) of the patients were diagnosed as having AIS by neurology residents. The mean age of the patients was 69 years (range 19–98) and 246 (47.8%) patients were male and 269 (52.2%) female. Stroke risk factors are shown in Table 1.

**Table 1.** Prevalence of Stroke Risk Factors

Risk Factor	No. (%) (n = 515)
**Hypertension**	341 (66.5)
**Diabetes mellitus**	91 (17.7)
**Hyperlipidemia**	48 (9.4)
**Coronary artery disease**	50 (9.8)
**Smoking**	82 (16.3)
**Arterial fibrillation**	97 (18.9)

A total of 159 (31.3%) patients arrived at the hospital in the first three hours of symptom onset, and 98 (19.1%) arrived at the hospital within the first two hours of symptom onset. Exclusion criteria other than time are shown in Table 2.

**Table 2.** Exclusion Criteria Other Than Time for r-TPA in Patients With Acute Ischemia^a^

	No. (%)
**Clearing spontaneously**	67 (13.1)
**Minor stroke symptoms (NIHSS score < 5)**	98 (19.1)
**Major deficit (NIHSS score > 22)**	39 (7.6)
**History of recent trauma or ischemic stroke (< 3 months)**	16 (3.1)
**Myocardial infarction in the previous 3 months**	3 (0.6)
**Recent major surgery (< 14 days)**	1 (0.2)
**SBP > 185 or DBP > 110**	69 (13.5)
**INR > 1.7**	17 (3.4)
**aPTT > 40 sec**	35 (7.1)
**Platelet < 100,000/mm** ^**3**^	12 (2.4)
**Glucose < 50 mg/dL**	2 (0.4)
**Seizure at onset**	21 (4.1)
**Hypodensity > 1/3 of cerebral hemisphere**	37 (7.2)

^a^ Abbreviations: aPTT, activated partial thromboplastic time; DBP, diastolic blood pressure; INR, international normalized ratio; NIHSS, national institute of health stroke scale; SBP, systolic blood pressure; TPA, tissue plasminogen activator.

For all the patients, the mean time interval between onset of symptoms and hospital arrival was 916 minutes (15.2 hours), hospital arrival and completed CT scan was 91 minutes, hospital arrival and completed investigations (also named door-to-needle time) was 147 minutes. The time interval between onset of symptoms and hospital arrival among patients who were eligible for thrombolytic therapy was 117 minutes. The time between symptoms onset and completed investigations was less than 180 minutes in 29 (5.7%) patients. Among 159 patients who arrived within 3 hours of symptom onset, 131 (82.3%) patients missed thrombolytic therapy due to delayed performance of brain CT scanning and laboratory tests and 38.3% (61/159) due to having other TPA contraindications. Some patients had both measures. Out of the remaining 16 patients (8 males and 8 females) were eligible for thrombolytic therapy; these patients comprised 10.0% of those who arrived early and 3.1% of all cases. Without considering time as a barrier for implementation of thrombolytic therapy, 288 (56.5%) of all patients could have been eligible for thrombolytic therapy.

## 5. Discussions

Although the safety and efficacy of thrombolytic therapy has been clearly demonstrated, the rate of its use in developing countries is still low (9, 11, 13, 14, 16). The main barriers for implementation of thrombolytic therapy in developing countries are:

- Delay in arrival of the patients to the emergency department after symptom onset, mainly due to poor recognition of stroke symptoms among the public and lack of rapid transportation to the hospital.
- Financial constraints; due to the high cost of the relevant drug.
- Lack of proper treatment facilities.
- Physicians’ fear of serious side effects of the drug, especially intracranial hemorrhage.
- Shortage of facilities in rural areas.
- Physicians’ lack of confidence in the efficacy of thrombolytic therapy (13, 14, 17).

In our study, 3.1% of patients were eligible for thrombolytic therapy. This is far lower than the percentage seen in developed countries which is mostly more than 10% (3, 4, 18), but similar to the other developing countries (9, 11, 13, 14, 16). Time between onset of symptoms and arrival at the hospital was similar to other studies (9, 14, 17, 19), but the time taken for in-hospital evaluations, including hospital arrival to completion of the CT scan, and door-to-needle time, was much longer in our center than in the hospitals of other developing countries (9, 14, 17, 19). According to the AHA guidelines, a CT scan should be completed within 25 minutes of the patient’s arrival in the emergency department (3). Some centers in India have successfully achieved this goal (17, 19), but in our center it took 91 minutes on average, which is far too long. Similarly, door-to-needle time should be less than 60 minutes, but in our study the average time was 147 minutes, which was longer than what is seen in other developing countries (9, 14, 17, 19). The rate of patients arriving at the hospital in the first three hours of the symptom onset (31.3%) was similar to one center in India (20), but was higher than other hospitals (12-14). Without considering time as an exclusion criteria, 56.5% of our patients could have been eligible for the thrombolytic therapy, a result similar to or higher than the results for centers in developed countries. Only 10.0% of patients who arrived early were eligible for thrombolytic therapy, which was far lower than the other centers (9, 13).

Our findings suggest that the rate of patients arriving early at the hospital after onset of symptoms, compares favorably with other reports and those contraindications for thrombolytic therapy outside of measuring time are similar to those of the other countries, even developed countries. In our hospital, the major barriers to implementing thrombolytic therapy for acute ischemic stroke patients were in-hospital delays, like initial patient assessment, CT scanning and running laboratory tests. There were two major limitations to this study. One of the most important barriers to thrombolytic therapy in developing countries is financial constraints due to the high cost of this therapy (10, 13, 20). Health insurance systems in most countries provide members with either no coverage or very limited coverage for thrombolytic therapy (10, 13, 14). There are some exceptions, for example; Thailand fully covers its population for this therapy (9). However, in one study in India, all seven patients who were eligible for thrombolytic therapy could not be treated due to the high cost of the drug (20). In Iran, thrombolytic therapy costs $1200 US for a 70 kg patient, a figure that is more than double of the country’s total expenditure on health per capita. Because we were not going to administer the drug to our patients, we could not assess whether they could afford the drug expense. However, in another study in the Northeast of Iran, Ghandehari et al. showed that just 30% of eligible patients were capable of paying for the TPA expense by themselves (10). We believe that the serious side effects of the treatment will cause a certain percentage of patients to refuse to sign the consent form and will thus not receive the treatment. This will further reduce the percentage of eligible patients. Our results suggest that we need to create an independent process for identifying patients with acute stroke symptoms, giving them the high priority in triage, CT scanning and running laboratory analysis and then fast tracking the patients through the system. A stroke educational program for the public is also essential, to help raise awareness of stroke symptoms and to encourage the public to seek medical care promptly.
